# Advances in single-cell omics and multiomics for high-resolution molecular profiling

**DOI:** 10.1038/s12276-024-01186-2

**Published:** 2024-03-05

**Authors:** Jongsu Lim, Chanho Park, Minjae Kim, Hyukhee Kim, Junil Kim, Dong-Sung Lee

**Affiliations:** 1 Department of Life Science, University of Seoul, Seoul, 02504 Republic of Korea; 2 School of Systems Biomedical Science, Soongsil University, Seoul, 06978 Republic of Korea

**Keywords:** PCR-based techniques, Next-generation sequencing

## Abstract

Single-cell omics technologies have revolutionized molecular profiling by providing high-resolution insights into cellular heterogeneity and complexity. Traditional bulk omics approaches average signals from heterogeneous cell populations, thereby obscuring important cellular nuances. Single-cell omics studies enable the analysis of individual cells and reveal diverse cell types, dynamic cellular states, and rare cell populations. These techniques offer unprecedented resolution and sensitivity, enabling researchers to unravel the molecular landscape of individual cells. Furthermore, the integration of multimodal omics data within a single cell provides a comprehensive and holistic view of cellular processes. By combining multiple omics dimensions, multimodal omics approaches can facilitate the elucidation of complex cellular interactions, regulatory networks, and molecular mechanisms. This integrative approach enhances our understanding of cellular systems, from development to disease. This review provides an overview of the recent advances in single-cell and multimodal omics for high-resolution molecular profiling. We discuss the principles and methodologies for representatives of each omics method, highlighting the strengths and limitations of the different techniques. In addition, we present case studies demonstrating the applications of single-cell and multimodal omics in various fields, including developmental biology, neurobiology, cancer research, immunology, and precision medicine.

## Single-cell omics: revolutionizing molecular profiling with high-resolution insights

Each cell is a bustling city of genetic material and proteins, but traditional methods often blur the individuality of each cell by averaging the data. Single-cell omics techniques, however, allow us to focus on individual cells, revealing the rich variety of cell types, states, and rare populations that make up our tissues and organs. In this review, researchers delve into the details of single-cell omics and multi-modal omics. They explore on how to isolate cells, add unique barcodes to keep track of them, and then analyze their genetic and molecular content. The integration of data across different molecular dimensions is a leap forward in the field, and it paves the way for future discoveries that could transform medicine. The potential implications of this research are vast, offering hope for more precise and personalized medical treatments in the future.

This summary was initially drafted using artificial intelligence, then revised and fact-checked by the author.

## Introduction

Single-cell omics techniques have transformed biological research, offering unprecedented insights into cellular intricacies. Conventional bulk sequencing methods have a limited ability to uncover heterogeneity and complexity within a population of cells, as they provide averaged data. In contrast, single-cell sequencing enables the characterization of individual cells, revealing diverse cell types, dynamic cellular states, and rare cell populations that were concealed within the ensemble of bulk measurements.

Single-cell analysis driven by high-throughput sequencing and mass spectrometry provides high-resolution insights into genomes, transcriptomes, proteomes, and epigenetics. This approach uncovers hidden complexities in cellular landscapes, providing novel insights into cellular development, diseases, and cellular mechanisms.

However, biological systems are complex and driven by interactions between omics layers. In recognition of this, the emerging field of single-cell multimodal omics has emerged to integrate information across diverse molecular dimensions within a single cell. This approach provides a holistic view of processes, illuminating the interconnected networks that shape cell behavior.

Multimodal omics data enable the identification of causal relationships between layers, revealing how genetics affect gene expression, epigenetics, proteins, and metabolites. This integrative approach is particularly useful for dissecting complex diseases. Understanding the molecular mechanisms underlying disease pathogenesis requires a multidimensional perspective.

In this review, we discuss the key principles and technical considerations underlying single-cell omics and multimodal omics. We explore the technical principles, experimental workflows, and biological insights gained from these approaches. Additionally, we highlight the challenges and opportunities in the field, discuss emerging technologies, and provide insights into the future directions of single-cell and multimodal omics research.

## Single-cell isolation and barcoding

Because single-cell sequencing technologies aim to understand and profile each cell according to its characteristics, cells must be separated and isolated from cell samples or whole tissues before analysis. Magnetic-activated cell sorting (MACS)^1^, fluorescence-activated cell sorting (FACS)^2^, and various microfluidic technologies are often employed for high-throughput studies. These technologies allow the isolation and analysis of large populations of cells in a more efficient and automated manner, enabling high-throughput experiments. FACS can simultaneously analyze cells according to size, granularity, and fluorescence, allowing multiparameter analysis^2^. Although FACS has become a prominent single-cell isolation method because of its ability to analyze multiple parameters simultaneously and its high specificity, it has certain technical limitations. For example, FACS requires sufficient cell density for effective analysis and may face challenges in isolating single cells from low-density populations. Rapid flow and fluorescence exposure during FACS can affect cell viability, potentially resulting in separation failure.

Microfluidic devices have revolutionized single-cell analysis by enabling the high-throughput processing of tens of thousands of single cells. These devices consist of microfluidic channels and reaction chambers controlled by valves, allowing for precise manipulation and isolation of droplets within microfluidic chips, where each droplet encapsulates a single cell, enabling the parallel processing of numerous cells. Another technology utilizes devices with nanowells that provide individual compartments for single cells. Microfluidic devices offer significant advantages, including increased throughput, reduced cost per cell, and the ability to scale down reaction volumes to the nanoliter or picoliter scale. This reduction in reaction volume minimizes reagent consumption and can shift the main cost barrier from library preparation to sequencing. Consequently, researchers can generate larger datasets while optimizing costs^3^.

Cell barcoding is a crucial step in a single-cell sequencing workflow, allowing libraries from multiple individual cells to be sequenced together in a single pool. This enables the efficient sequencing of many cells while preserving their identity for downstream analysis. In plate-based techniques, the cell barcode is typically added to the final PCR step before sequencing, in which sequencing libraries from different cells are combined. Microfluidics-based barcoding methods offer the advantage of incorporating cell barcodes into the protocol earlier, often allowing the entire pool of libraries to be processed in a single tube. This early incorporation of barcodes reduces the number of handling steps and the potential sample loss^4^.

Cell barcoding techniques offer a powerful means to barcode and sequence individual cells, enabling the deconvolution of sequence data into files that represent each cell. These methods facilitate high-throughput single-cell sequencing while preserving cell identity and enabling downstream analysis.

## Objectives of single-cell mono-omics

### Genome

Single-cell genomics provides a new perspective on biological functions through the study of genetic variants at the individual cell level. However, the small amount of DNA obtained from a single cell (typically at the picogram level) poses challenges for amplification and analysis. Genomic research has progressed rapidly in recent decades with the development of amplification methods. Whole-genome amplification (WGA) technologies have been developed to amplify fragments of the entire genome of a single cell while minimizing amplification errors and avoiding contamination.

One commonly used technique is degenerate oligonucleotide-primed (DOP)-PCR, which is a specialized PCR method for WGA. DOP-PCR utilizes primers with random sequences to bind to various sites in the genome, followed by a second amplification stage in which fragments tagged by a specific sequence are selectively amplified^5^. Although DOP-PCR allows amplification from low amounts of starting materials, it can result in low genome coverage owing to site-specific preferential amplification. Multiple displacement amplification (MDA) amplifies DNA isothermally using φ29 DNA polymerase, resulting in high coverage but exhibiting amplification bias^6^. However, methods that rely on WGA produce artifacts that complicate the discovery of variants. These artifacts include locus and allelic dropouts, uneven amplification, chimeric DNA molecules, and base-copy errors.

Primary template-directed amplification (PTA) is a recently reported method that builds on MDA to achieve quasilinear amplification. By incorporating exonuclease-resistant terminators, PTA suppresses additional amplification, resulting in higher accuracy, uniformity, and reproducibility than other methods for single-cell genome analysis^7^. Multiplexed end-tagging amplification of complementary strands (META-CS) allows the accurate identification of de novo single-nucleotide variants (SNVs) in a single cell. This enables amplification in a one-tube reaction while labeling the two DNA strands differently, facilitating the comparison of complementary positions and filtering out false positives^8^.

Microfluidic-based WGA methods are promising and are actively being developed. These methods offer numerous advantages over traditional WGA techniques, making them highly attractive for single-cell genomic research. Microfluidic platforms provide a high degree of automation and integration, allowing multiple steps in the WGA process to be performed within a single device. This integration simplifies workflow, saves time, and minimizes the risk of sample contamination or loss^3^.

Single-stranded sequencing using microfluidic reactors (SISSOR)^9^ utilizes a microfluidic device to isolate DNA in a single cell. The DNA is then separated into Watson and Crick strands, which are randomly partitioned into nanoliter-scale compartments within the device. This partitioning allows for the amplification and barcoded library preparation of genomic DNA. SISSOR offers high sequencing accuracy with low error rates, although it may exhibit reduced genomic coverage owing to the potential loss of DNA fragments during the strand separation and partitioning processes.

### Transcriptome

The transcriptome directly influences protein translation. Although the genetic information is nearly identical across all human cells, the transcriptomic data of individual cells reveals distinct gene activity patterns. Recent advances in single-cell RNA-seq have enabled the characterization of cells at the single-cell and spatiotemporal levels and the establishment of diverse projects, such as the Human Cell Atlas project^10^. In addition to characterization, scRNA-seq has been used to study cytidine deaminase (CDA) as a potential druggable target in *ALK* fusion-positive non-small cell lung cancer (NSCLC)^11^.

Cell expression by linear amplification and sequencing2 (CEL-seq2) enhances CEL-seq using barcodes that incorporate UMIs and random priming, leading to improved read mapping percentages and sensitivity while reducing bias^12^. In massive parallel single-cell RNA sequencing (MARS-seq2.0), the reaction volume is reduced during reverse transcription, resulting in decreased noise and increased sensitivity^13^. However, these methods primarily capture 3′ end transcripts, making it difficult to determine sequences or isoforms at the 5′ end^13^.

Droplet-based technologies such as 10X Genomics Chromium^14^ and Drop-seq^15^ have become popular owing to their cost-effectiveness and high throughput. Beads are used to capture RNA from the oil and create reaction droplets. Chromium uses soft hydrogel beads, whereas Drop-seq uses small hard resin beads, resulting in different cell capture rates.

Split pool ligation-based transcriptome sequencing (SPLiT-seq) involves iterative splitting and pooling of cells, allowing for diverse cell barcode combinations^16^. This method accommodates fixed cells or nuclei and offers flexibility in experimental design^16^.

Several methods, including molecular crowding single-cell RNA barcoding and sequencing (mcSCRB-seq)^17^, switching mechanism at 5′ end of RNA template sequencing3 (SMART-seq3)^18^, and FLASH-seq^19^, employ full-length cDNA library construction and sequencing. These methods utilize template-switching oligos (TSOs) to create full-length cDNA libraries and identify the 5′ ends of transcripts, and they incorporate UMIs to mitigate PCR bias. FLASH-seq combines split reverse transcription and PCR processes with an improved reverse transcriptase, enhancing cDNA yield and reducing amplification noise and hands-on time^19^.

Vast transcriptome analyses of single cells using dA-tailing (VASA-seq)^20^ are available in both plate-based and droplet-based formats, providing versatility based on experimental objectives. This approach enables the formation of full-length cDNA libraries and the capture of nonpolyadenylated transcripts. VASA-seq can detect coding RNAs, long noncoding RNAs, transcription factors, and small noncoding RNAs^20^.

All of the abovementioned scRNA-seq methods have constraints in capturing longer transcripts, identifying splicing events, and distinguishing between transcript isoforms because they are based on short-read sequencing. Specialized methods have been developed to address these limitations. Multiplexed array isoform sequencing (MAS-ISO-seq) employs long-read sequencing by attaching a dU-containing adapter to cDNA ends, enabling the sequencing of long reads in a single pass^21^. Single-nucleus isoform RNA sequencing (SnISOr-seq) distinguishes intronic reads by splitting transcripts into intronic and exonic cDNAs and analyzing only exonic cDNA through long-read sequencing, thereby enhancing the analysis of the desired exons^22^. Long-read sequencing-based methods such as MAS-ISO-seq and SnISOr-seq address the limitations of short-read sequencing-based methods and offer improved capabilities for characterizing longer transcripts and transcript isoforms^23^. The properties of these methods are listed in Table 1.

**Table 1:** Comparison of the properties of transcriptome sequencing methods.

Method	Sorting	UMI	Transcriptome amplification	Region	Read length	Published year	Reference
CEL-seq2	Fluidigm C1	O	IVT	3′ end	Short read	2016	^12^
Mars-seq2	FACS	O	IVT	3′ end	Short read	2019	^13^
10X Genomics	Droplet	O	PCR	3′ end	Short read	2017	^14^
mcSCRB-seq	FACS	O	PCR	3′ end	Short read	2018	^17^
SMART-seq3	FACS	O	PCR	Full length	Short read	2020	^18^
SPLiT-seq	FACS	O	PCR	3′ end	Short read	2018	^16^
FLASH-seq	FACS	O	RT‒PCR	Full length	Short read	2022	^19^
VASA-seq	FACS or Droplet	O	IVT	Full length	Short read	2022	^20^
MAS-ISO-seq	FACS	O	PCR	Full length	long read	2021	^21^
SnISOr-seq	Droplet	O	linear/asymmetric PCR	Full length	long read	2022	^22^

### Proteome

Proteome research at the single-cell level provides a wealth of information about the diversity of proteins within a cell population and important insights into cellular functions, disease mechanisms, and developmental processes because proteins can be considered the end material of biological processes in cells.

Mass spectrometry (MS) allows researchers to identify and quantify proteins based on their mass-to-charge ratios (m/z). MS-based methods can be used for single-cell protein sequencing by coupling with other techniques to capture and isolate individual cells.

Matrix-assisted laser desorption/ionization (MALDI)^24^ and laser ablation electrospray ionization (LAESI) are MS-based techniques commonly used for single-cell protein sequencing. In MALDI, a laser is used to ionize proteins on a matrix-coated surface, which are then detected using a mass spectrometer. In LAESI, a laser is used to ablate proteins directly from the cell surface, after which the proteins are ionized and detected using a mass spectrometer. These techniques offer high sensitivity and are well suited for analyzing small numbers of cells^24^.

Fluorescence-based methods are another approach to single-cell protein sequencing. These methods rely on fluorescent probes or antibodies that specifically bind to the proteins of interest. The fluorescence signal is detected and quantified using microscopy or flow cytometry. Single-cell western blotting (scWB)^25^ is a fluorescence-based technique that allows the detection of specific proteins within individual cells. In scWB, a single cell is lysed, and the proteins within the cell are subsequently separated via gel electrophoresis. The separated proteins are then transferred onto a membrane as in traditional western blotting.

### Methylome

DNA methylation is an epigenetic modification involving the addition of CH_3_ to deoxyribonucleosides, predominantly 5-methylcytosine (5mC), that is particularly common in vertebrates. DNA methyltransferases (DNMTs) deposit and maintain methyl groups, whereas ten-eleven translocation (TET) dioxygenase removes them. CpG islands (CGIs), often found in promoters and gene bodies, are primary sites of methylation. CpG promoter methylation typically reduces gene expression, whereas gene body methylation enhances gene expression. Methylation rarely occurs at non-CpG (CpH) sites, which are frequently found in neurons.

Methylation plays a role in X-chromosome inactivation, genomic imprinting, and transposon suppression. It is also an epigenetic feature of cancers and other diseases. Therefore, many methylation assays (bisulfite sequencing, chromatography, mass spectrometry, ELISA, and restriction digestion) have been developed. Here, we describe single-cell-based methylome methods (Table 2).

**Table 2:** Methylome sequencing methods with brief explanations and differences in workflows.

Method	Protocol Base	Sorting	Average CpG Coverage	Comments	Refs
scRRBS	RRBS	Mechanical separation	1.02 × 10^6^ (40% of total 2.5 × 10^6^)	Integrated all steps in single-tube reaction without including any purification steps prior to the bisulfite treatment step	^22^
scBS	Bisulfite treatment	Flow cytometry	3.7 × 10^6^	First method to use postbisulfite adapter tagging	^26^
scWGBS	Bisulfite treatment	FACS	~ 10^6^	Used postbisulfite adapter tagging	^28^
MID-RRBS	RRBS	FACS	3.5 ~ 23.1 × 10^4^	Conducted RRBS method on a microfluidic device	^36^
sn-mC-seq	Bisulfite treatment	FANS	22.2 ± 5.79% (Genome wide coverage)	Single nuclei-based method/used random priming	^30^
sn-mC-seq2	Bisulfite treatment	FANS	~30.8 ± 7.5% (Genome wide coverage)	Used RP-H (H = A, T, C) random primers for destabilizing primer hybridization, incorporated dephosphorylation step to inactivate dNTP	^31^
sci-MET	Bisulfite treatment	FANS	0.05 ~ 7% (human)	Used combinatorial indexing	^33^
sci-METv2	Bisulfite treatment	FANS	~2.2 × 10^6^(LA) ~0.3 × 10^6^(SL) (human)	Two improved versions of sci-MET: sciMETv2.LA (linear amplification, accuracy), sciMETv2.SL (splint ligation, speed)	^34^
scSPLAT	Bisulfite treatment	FACS	At most 20 ~ 40% of human cell	Used RP-H for second strand synthesis and used splinted dsDNA adapters before PCR step	^32^
Msc-RRBS	RRBS	FACS (By cell cycle)	~0.9 × 10^6^ (Human)	Inline barcode single-well enzymatic reaction	^29^
sci-EM	Enzymatic conversion	FANS	Not specified	Combined combinatorial indexing with enzymatic conversion	^35^

Single-cell bisulfite sequencing (scBS-Seq) was adapted from traditional bisulfite sequencing for single-cell DNA methylation analysis at a single-base resolution^26^. DNA bisulfite treatment converts unmethylated cytosines to uracil while preserving methylated cytosines^26^. To mitigate the high costs of whole-genome sequencing in scBS-Seq, technologies such as scRRBS have focused on CpG-enriched genomic regions^27^. In scRRBS, the process has been streamlined in a single tube^27^.

However, because adapters are ligated before bisulfite treatment, these methods suffer from high DNA loss. In single-cell whole-genome bisulfite sequencing (scWGBS) and scBS, this problem is solved via postbisulfite adapter tagging (PBAT)^26,28^. Unlike scWGBS or scBS, Msc-RRBS does not utilize postbisulfite adapter tagging (PBAT) and is not affected by bisulfite treatment because of the use of a methylated adapter^29^.

Single-nucleus methylome sequencing 2 (SnmC-seq2), an improved version of snmC-seq^30^, reduced the frequency of hybridization of random primers by using random primer H (RP-H), which lacks the nucleotide ‘G’. This reduces the dNTP contamination rate through additional quenching steps^31^. Single-cell splinted ligation adapter tagging (scSPLAT) improves mappability using a splinted adapter as the second adapter. No artificial low-complexity sequence is added, and the process is free from the risk of artificial sequences due to the carryover of free nucleotides^32^.

Single-cell combinatorial indexing for methylation analysis (sci-MET) is a scWGBS method that incorporates a combinatorial indexing strategy^33^. Furthermore, an enhanced version of sci-MET, sci-METv2^34^, exhibits high methylome coverage for sci-METv2.LA (linear amplification) and reduced costs and preparation time for sci-METv2.SL (splint ligation). However, it has a low read insert size due to damage during bisulfite conversion, which can be addressed using enzymatic conversion methods^34^. A recently developed method, single-cell combinatorial indexing with enzymatic conversion (sciEM), addresses the challenges associated with bisulfite conversion by utilizing *APOBEC, TET2* for enzymatic conversion, and a G-depleted random linear primer to improve CpH mapping^35^. Compared with previous methods, sciEM provides increased genomic coverage^35^. Enzymatic conversion significantly improves DNA methylation^35^.

Microfluidic diffusion-based RRBS (MID-RRBS) is a methylation sequencing method that utilizes a reagent-swapping approach to reduce the loss of DNA molecules during the purification step between bisulfite treatment and desulfonation^36^.

### Chromatin accessibility

Nucleosomes, which consist of DNA wrapped around histone proteins, hinder transcription, replication, repair, and recombination by obstructing relevant factors. Therefore, chromatin accessibility sequencing techniques rely primarily on enzymatic methylation or cleavage. Single-cell adaptations of these methods enable examination of chromatin accessibility at the individual cell level, providing insights into the dynamics and heterogeneity of chromatin accessibility.

The discovery of periodic DNase hypersensitivity sites (DHSs) has played a crucial role in the development of genome-wide chromatin accessibility sequencing methods. scDNase-seq detects a greater number of DHSs per cell than scATAC-seq, thereby providing increased resolution. However, this method requires a longer hands-on time^37^. scMNase-seq is a single-cell adaptation of MNase-seq that utilizes MNase as both an endonuclease and an exonuclease^38^. Unlike other methods, scMNase-seq has the advantage of cutting linker DNA, allowing the precise determination of nucleosome boundaries^38^. However, this method provides limited information, capturing only 3%–10% of the nucleosome and subnucleosomal fragments^38^.

Single-cell nucleosome occupancy and methylome sequencing (scNOMe-seq) utilizes CpG methyltransferases to analyze accessibility by detecting methylation levels^39^. Unlike other methods that rely on read counting, scNOMe-seq provides detailed accessibility information by analyzing the methylation status of individual sequenced reads, including CpG sites that independently report accessibility^39^. In single-cell variants, the ability to simultaneously detect chromatin accessibility and methylation levels makes this approach a valuable tool for studying the heterogeneity of single cells within complex mixtures^39^.

Tn5 transposase-based methods such as single-cell assays for transposase-accessible chromatin using sequencing (scATAC-seq) have revolutionized single-cell epigenomic mapping^40^. High-throughput scATAC-seq utilizes fluorescence imaging and addressable reagent deposition to achieve high throughput and cost effectiveness^41^. Plate-based scATAC-seq minimizes material loss and labor while generating highly complex data^42^. Droplet microfluidics-based approaches such as dscATAC-seq and dsciATAC-seq further enhance cell throughput and barcoding capabilities by utilizing combinatorial indexing^43^.

### Chromatin conformation capture

In humans, the nucleus contains chromosomes that are organized into territories. These territories comprise different compartments, including topologically associated domains (TADs) and DNA loops. The CCCTC-binding factor (CTCF) shapes these features, affecting gene expression and genome organization in 3D. Chromosome structure and enhancer–promoter contacts affect gene expression. An altered chromatin conformation can lead to disease. Recent studies have shown that structural variants (SVs) that affect 3D genome organization contribute to cancer and other disorders^44^.

To investigate chromatin conformation and understand its heterogeneity and dynamics, several techniques based on Hi-C have been developed, which reduce the sequencing library size by utilizing a pull-down step^45^. Hi-C and its derivatives can be classified into two groups depending on their cyclization system, dilute ligation systems and in situ ligation systems, for all single-cell Hi-C protocols, such as Dip-C^46^ and single-cell Hi-C^47^.

Single-cell high-throughput chromosome conformation capture (scHi-C) is a powerful method for investigating the 3D structure of an entire genome in individual cells. This method provides insight into the folding structure of the genome in a single cell at specific time points. The first single-cell Hi-C (scHi-C) technique introduced in-nucleus ligation, in which proximity ligation was performed within intact nuclei rather than after nuclear lysis, as in dilute ligation-based Hi-C^47^. This in-nucleus ligation approach improved the quality of the Hi-C data by preserving the chromatin conformation within individual cells. However, this method still requires mechanical isolation of single nuclei within individual cells. The introduction of combinatorial indexing-based methods has solved this problem. One such method is sciHi-C, which utilizes combinatorial indexing to process thousands of cells without physical isolation or microfluidic manipulation^48^. Another combinatorial indexing-based method, called single cell-indexed DLO Hi-C (sciDLO Hi-C), which does not require biotin labeling or pulldown, has also been developed^49^.

### Histone modification

Histone modifications play crucial roles in regulating gene expression. Various chemical groups can be added to and removed from the N-terminal tails of histones. Histone modifications result in different chromatin states that can activate or repress gene expression. Understanding the landscape of histone modifications at the single-cell level is essential for studying epigenetic programs and predicting transcription states.

Chromatin immunoprecipitation (ChIP) is used to identify the DNA-binding sites of a specific protein. After fragmentation with micrococcal nuclease (MNase), histone–DNA complexes containing specific modifications are immunoprecipitated using specific antibodies. ChIP-seq requires a large number of samples because of its low signal-to-noise ratio (SNR). Droplet-based chromatin immunoprecipitation (Drop-ChIP) overcomes the limitation of labeling the target loci of chromatin to capture histone modifications at the single-cell level^50^.

Another strategy for reducing noise is cleavage of targets and release using nucleases (CUT and RUN)^51^. After binding to target histones, MNase binds to protein A and specific antibodies to cleave and release chromatin fragments. CUT and RUN requires additional steps, such as DNA end polishing and adapter ligation, for sequencing library preparation, which increases time, cost, and labor. A strategy using Tn5 transposase was developed to address these problems. Single-cell cleavage under targets and tagmentation (scCUT and tag) uses the fusion protein pA-Tn5, which binds to antibodies^52^. Preloaded DNA adapters in Tn5 are integrated into chromatin, and the indexed DNA fragments are released at the same time.

Most existing methods for mapping histone marks are limited to profiling one histone modification at a time. Single-cell chromatin immunocleavage and unmixed sequencing (scChIX-seq) allows for the analysis of multiple histone markers in a single cell. We analyzed two histone markers, both together and separately. Two histone signals from double-incubated cells were separated and interpreted using single-incubated datasets as training data to profile each histone marker individually via a computer algorithm^53^.

### Single-cell multimodal omics

**Fig. 1:**
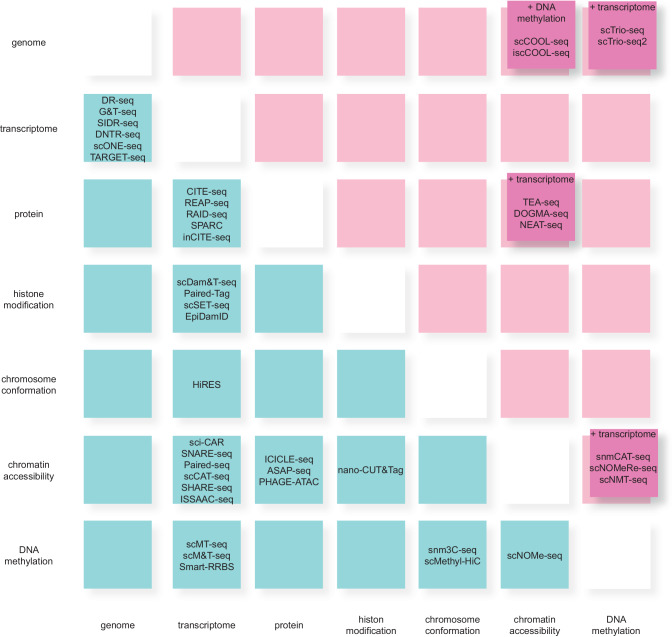
Sequencing methods for single-cell multimodal omics analysis. Numerous single-cell multiomics sequences have been developed and can be classified into seven types. The blue box represents dual omics technology, and the magenta box represents technology that handles three or more omics simultaneously.

Single-cell multimodal omics techniques have emerged as powerful tools for studying complex biological processes occurring within single cells. These techniques enable the simultaneous analysis of multiple omics layers, such as genomics, transcriptomics, proteomics, and epigenomics, within individual cells. By integrating information from different molecular levels, researchers can gain a more comprehensive understanding of cellular behavior and regulation. There are several variations of single-cell multimodal omics techniques, each focusing on different molecular layers (Fig. 1).

### Single-cell multimodal omics methods that simultaneously address the genome and transcriptome

Simultaneous examination of the genome and transcriptome is an important experimental method for directly identifying changes in phenotypic material because the transcriptome is generated from the genome through transcription. The observation of mutations and specificity in the reconstitution of DNA sequences into RNA sequences is an important indicator of phenotypic changes in a cell.

**Fig. 2:**
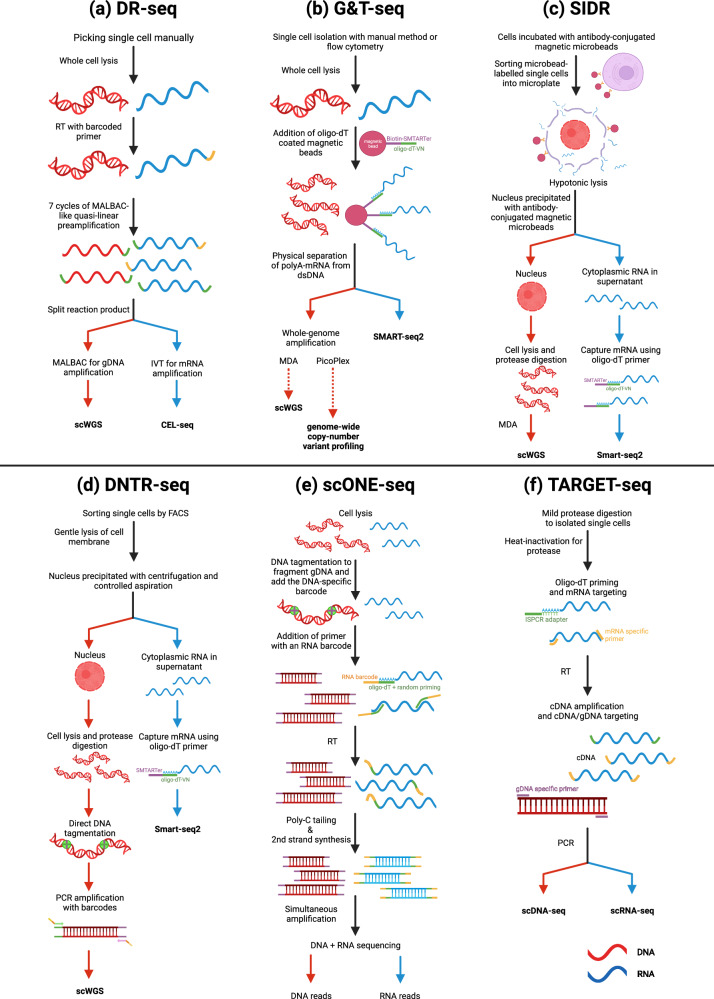
Protocols for multimodal omics methods for simultaneous analysis of the genome and transcriptome. Here, we present an overview of single-cell multimodal omics methods that coprofile the genome and transcriptome. **a** gDNA-mRNA sequencing (DR-seq) involves preamplification of DNA and RNA from a single cell before splitting the material for separate genome and transcriptome sequencing. **b** In genome and transcriptome sequencing (G&T-seq), the genetic material is amplified after physical separation using beads. **c** Simultaneous isolation of genomic DNA and total RNA (SIDR-seq) involves the use of magnetic microbeads for hypotonic lysis to isolate the nucleus from the cytoplasmic RNA. **d** Direct nuclear tagmentation and RNA sequencing (DNTR-seq) gently lyses the cell membrane, enabling the precipitation of the nucleus through centrifugation and controlled aspiration, which separates the intact nucleus from the RNA. **e** For scONE-seq, all processes, including amplification and sequencing, are performed in single tubes. This is possible because genomic DNA and RNA are barcoded differentially. **f** Sample preparation for TARGET-seq relies on mild protease digestion, which enhances the release of genomic DNA and mRNA. Separate amplification processes are used for DNA and RNA analyses. For each process, red lines represent DNA- and DNA-related processes, whereas blue lines represent RNA- and RNA-related processes.

The earliest methods of this type were gDNA-mRNA sequencing (DR-seq)^54^ and genome and transcriptome sequencing (G&T-seq)^55^, which require dividing RNA and DNA extracted from a single cell. In DR-seq, preamplified nucleic acids are divided into RNA and DNA and sequenced (Fig. 2a). DR-seq is a plate-based and low-throughput method that minimizes the risk of nucleic acid loss. In G&T-seq, oligo-dT-coated magnetic beads are used to separate poly-A mRNA from DNA, and the fractionated DNA and RNA are analyzed (Fig. 2b). However, DR-seq is limited by the fact that only the 3′ end of RNA can be sequenced, while G&T-seq can sequence the full length of RNA.

While simultaneous isolation of genomic DNA and total RNA sequencing (SIDR-seq)^56^ and direct nuclear tagmentation and RNA-sequencing (DNTR-seq)^57^ share similarities with G&T-seq in terms of amplifying genetic materials after separation, they differ from G&T-seq in that they separate intact nuclei. For SIDR-seq, cells are first cultured with antibody-conjugated magnetic microbeads (Fig. 2c). This step allows selective labeling of target cells. Subsequent separation is achieved through hypotonic lysis, which causes cells to swell and rupture, resulting in the release of cytoplasmic RNA while preserving an intact nucleus. This approach is advantageous for studying nonpoly(A) RNA and long RNAs and for accurate detection of copy number variations (CNVs) and single-nucleotide polymorphisms (SNPs). In contrast, the separation process in DNTR-seq involves partial lysis of the cell membrane, followed by centrifugation and aspiration to separate intact nuclei from other cellular components (Fig. 2d).

TARGET-seq focuses on improving coverage of key mutations^58^. Sample preparation involves mild protease digestion to increase the release of gDNA and mRNAs, followed by heat inactivation of the protease to prevent interference in subsequent steps (Fig. 2f). RT and PCR amplification are performed separately to generate cDNA from mRNA and amplify the gDNA, respectively. In contrast, scONE-seq^59^ simplifies the measurement of single cells in a one-tube reaction. During sample preparation, gDNA and RNA are barcoded differentially. Differentially labeled gDNA and cDNA are simultaneously amplified and converted into a sequencing library in a single-tube reaction^59^ (Fig. 2e).

### DNA methylation-related methods for single-cell multimodal omics analysis

**Fig. 3:**
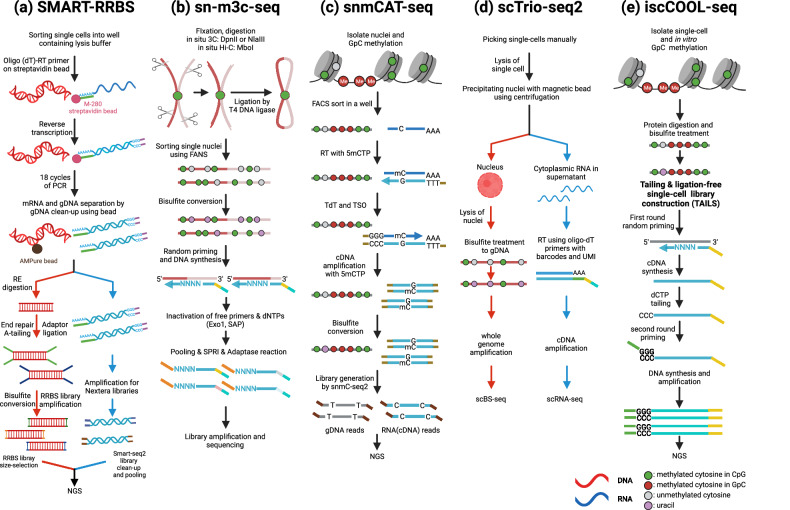
Representative multimodal omics protocols related to the methylome. Overview of five types of single-cell multimodal omics methods for analyzing diverse combinations of omics-containing methylomes. **a** Protocol of switching mechanism at 5′ end of RNA template-reduced representative bisulfite sequencing (SMART-RRBS) for the analysis of the transcriptome and methylome. DNA was isolated from RNA using AMPure beads. **b** Protocol for single-nucleus methyl chromosome conformation capture sequencing (sn-m3C-seq) for the analysis of the methylome and chromosome conformation. The biotin ligation step for selecting the ligated DNA is omitted to minimize the loss of DNA fragments. **c** Protocol for single-nucleus methylcytosine, chromatin accessibility, and transcriptome sequencing (snmCAT-seq) for the analysis of the methylome, chromatin accessibility, and transcriptome. This method does not mechanically separate DNA from RNA. Instead, DNA and RNA are separated during the data processing step by utilizing methylated cytosines in the cDNA synthesis step. **d** Protocol of single-cell triple omics sequencing 2 (scTrio-seq2) for the analysis of the genome, transcriptome, and methylome. The DNA and RNA are separated by centrifugation, and reverse transcription is performed using oligo-dT primers with barcodes and UMIs. **e** Protocol for improved single-cell multiomics sequencing (iscCOOL-seq) for the analysis of the genome, methylome, and chromatin accessibility. TAILS is used to construct the libraries. In (**a**, **b**, **d**), the red lines indicate DNA, and the blue lines indicate RNA. In (**c**, **e**), the gray lines indicate DNA.

Simultaneous examination of the transcriptome and methylome can provide valuable insights into the interplay between DNA methylation and transcription in cell populations with inherent heterogeneity. Single-cell genome-wide methylome and transcriptome sequencing (scM&T-seq) (the application of scBS-seq to G&T-seq), which physically separates RNA and DNA using oligo-dT-attached beads, has demonstrated a negative association between non-CGI promoter methylation and transcription in single cells^60^. Another method, scMT-seq (the combination of scRRBS with Smart-seq2), which separates the cytosolic fraction by micropipetting, revealed that methylation of CpG promoters has no effect on gene expression levels. scMT-seq offers higher transcriptome coverage than single-cell triple omics sequencing (scTrio-seq) but has low CpG coverage and a high rate of allele drop-out^61^. SMART-RRBS^62^ (the combination of MSC-RRBS with Smart-seq2), which divides DNA and mRNA using oligo-dT primers, can be used to identify rare tumor cells, study drug mechanisms, and detect CNVs^62^ (Fig. 3a). This method covers three times as many CpGs as scM&T-seq and generates fewer unwanted adapter-only sites than random-primer-based methods. However, this approach is more expensive than droplet or combinatorial indexing-based methods and has a lower throughput per cell, a low copy number of each genomic locus in a single diploid cell, and sparse methylome coverage^62^.

To fully understand the complex interactions and relationships between DNA methylation patterns and chromatin conformation, researchers have developed protocols that simultaneously analyze both DNA methylation and chromatin conformation.

sn-m3C-seq combines scHi-C with snmC-seq2^31^. The sn-m3C-seq protocol modifies the standard 3 C or Hi-C protocols by omitting certain steps (such as biotin fill-in and pull-down) to minimize data loss, particularly for methylation and ligation data (Fig. 3b). This modification results in a greater percentage of captured reads than other single-cell Hi-C methods^63^. Using sn-m3C-seq, researchers have defined different cell types within the human prefrontal cortex (PFC), demonstrating that contact maps can be used to distinguish between nonneuronal cells and neurons^64^. Moreover, sn-m3C-seq has been employed to investigate the variability between the methylome and chromosome conformation during brain development and to construct single-cell DNA methylomes and a 3D genome atlas of the mouse brain^65,66^. Given the significance of chromatin conformation and DNA methylation in various diseases, advancements of this methodology to include transcriptome analysis could offer a comprehensive understanding of holistic cellular responses at the individual level, particularly in diseases such as cancer.

Methyl-HiC is another method that can be used to simultaneously analyze the methylome and chromosome conformations^67^. Unlike sn-m3C-seq, methyl-HiC includes biotin ligation and pull-down steps^67^. Methyl-HiC is reported to detect 38,827 short-range contacts (<1 kb) and 77,811 long-range contacts (≥1 kb), whereas sn-m3C-seq can detect a significantly greater number of contacts (646,971 short-range and 195,160 long-range on average per cell)^68^. The observed discrepancy might be attributed to differences in the methodologies and protocols used for the two methods.

Simultaneous analysis of the genome, transcriptome, and DNA methylome is highly desirable in cancer research because of the substantial heterogeneity observed across these three omics layers. This demand has led to the development of a method called scTrio-seq that has been used to identify subpopulations of human hepatocellular carcinoma cells^69^ (Table 3). This method combines scRRBS, which detects DNA methylation and CNVs, with scRNA-Seq^69^. By integrating these techniques, researchers can investigate the positive correlation between DNA copy number and gene expression within relevant genomic regions and explore subpopulations with distinct CNVs, methylation levels, and RNA expression in hepatocellular carcinoma (HCC)^69^. scTrio-seq2, an improved version of scTrio-seq, incorporates multiplexed single-cell RNA-seq (scRNA-seq) using unique molecular identifiers (UMIs) inserted into oligo-dT primers for transcriptome profiling^70^ (Fig. 3d). This method also incorporates scBS-seq to profile DNA methylation levels across the entire genome. scTrio-seq2 has been used to analyze demethylation aspects and differences in methylation levels between normal and cancer cells^70,71^ (Table 3).

**Table 3:** Results obtained by using methylome sequencing methods (multimodal omics sequencing methods) and categories of those findings.

Categories	Method	Results	Ref
Development	scCOOL-seq	Chromatin conformation and DNA methylation undergo distinct changes that do not happen simultaneously following fertilization	^74^
	scNOMeRe-seq	Role of DNA methylation remodeling in reconstructing genetic lineages in early embryos	^86^
	scTrio-seq2	Slower genome demethylation in primitive endoderm cells than in epiblast and trophectoderm cells	^71^
	sn-m3C-seq	Remodeling of DNA methylation is temporally separated from chromatin state dynamics and mainly occurs during late-gestational to early-infant-development (In human frontal cortex and hippocampus)	^65^
	iscCOOL-seq	Integrating and analyzing of chromatin accessibility, DNA methylation and gene expression in growing mouth oocytes	^73^
	scM&T-seq	Correlations of methylation patterns of distal regulatory regions with gene expression (pluripotency factors)	^60^
	scNMT-seq	De novo methyltransferase is dispensable for major cell type development at E8.5 in mouse development but crucial for silencing prior or alternative cell fates such as pluripotency and extraembryonic programs	^98^
Brain	scMT-seq	Negative correlation between methylation in non-CGI promoters and gene expression in dorsal root ganglion neurons	^61^
	snmCAT-seq	Reconstruction of regulatory lineages for cortical cell populations Identification of distinct genetic risk enrichment associated with neuropsychiatric traits	^87^
	sn-m3C-seq	Constructing single-cell DNA methylome and 3D genome structure atlas of adult mouse brain	^66^
Senescence	scM&T-seq	Relation of aging with a global increase in transcription and methylation heterogeneity	^99^
Tumor	scTrio-seq	Identification of subpopulation of human hepatocellular carcinoma cells and cellular heterogeneity within a subpopulation	^69^
	scTrio-seq2	Differences in DNA methylation levels between primary and metastatic colorectal tumors are mainly caused by the different sublineage composition	^70^

The development of single-cell multiomics sequencing (scCOOL-seq) has enabled the simultaneous measurement of multiple epigenomic features in single cells^72^. This technique allows the analysis of CNVs, ploidy, DNA methylation, nucleosome positioning, and chromatin state within individual cells by combining PBAT-seq and NOMe-seq data^72^. scCOOL-seq can be used to study embryonic development and pathological conditions such as tumorigenesis, and it has been used to perform single-cell and parental allele-specific analyses in early mouse embryos^72^. An improved version of scCOOL-seq, iscCOOL-seq, was developed to increase the mapping rate^73^. By replacing PBAT with TAILS (a tailing- and ligation-free method for single cells), iscCOOL-seq achieved a higher mapping rate^73^ (Fig. 3e). This breakthrough has facilitated the examination of DNA methylation, chromatin accessibility, and gene expression^73,74^ (Table 3).

### Single-cell multimodal analysis for studying the interplay between epigenetic regulation and gene expression

Concurrent examination of the epigenome and transcriptome within individual cells allows the investigation of the relationship between epigenetic regulation and gene activity at the single-cell level, providing a deeper understanding of cellular heterogeneity and regulatory dynamics.

The sci-CAR method employs combinatorial indexing to merge sci-RNA-seq and sciATAC-seq, enabling concurrent analysis of the transcriptome and chromatin accessibility^75^. Despite its advantages in terms of throughput, sciCAR may yield sparse data, particularly concerning chromatin accessibility^75^. In contrast, scCAT-seq physically separates mRNA and DNA using Smart-seq2 for transcriptome analysis and scATAC-seq for chromatin accessibility analysis^76^. This approach successfully mapped chromatin accessibility and transcriptomes in early embryos^76^. SNARE-seq, a droplet-based method, captures both gDNA and mRNA, providing superior chromatin accessibility data compared with that of sci-CAR^77^. Paired-seq utilizes ligation-based combinatorial indexing to simultaneously barcode cDNA and gDNA, thereby increasing the throughput^78^. This method employs an amplify-and-split strategy to sequence cDNA and gDNA separately without the need for physical mRNA and gDNA separation^78^. SHARE-seq utilizes combinatorial indexing for barcoding and employs streptavidin beads to separate DNA and cDNA^79^. A recently developed method, ISSAAC-seq, combines SHERRY (single-cell chromatin accessibility profiling by integrating ATAC-seq and RNA-seq) and scATAC-seq and is suitable for both FACS and droplet-based methods^80^.

Advancements in DNA adenine methyltransferase identification (DamID) have facilitated simultaneous transcriptome and histone modification analyses. Single-cell DamID with mRNA sequencing (scDam and T-seq) merges single-cell DamID with CEL-seq2, probing DNA–protein interactions and transcription in individual cells^81^. By methylating adenines near the protein of interest, the *E. coli* Dam methyltransferase tags specific proteins, facilitating DNA–protein interaction investigations^81^. EpiDamID, an extension of DamID, overcomes the limitations of fusing dams with chromatin-binding modules specific to histone modifications^82^. This innovation allows for the profiling of various histone PTMs at a single-cell resolution, unveiling the interplay between gene expression and histone modifications at the cellular level^82^.

In addition, paired-tag and same-cell epigenome and transcriptome sequencing (scSET-seq) were used to coprofile histone modifications and transcriptomes^83^. The paired-tag method expands upon the paired-seq method, simultaneously allowing for the capture of open chromatin and gene expression information via the CUT&Tag strategy^83^. scSET-seq is also based on CUT&Tag and offers a similar approach^84^.

Single-cell nucleosome, methylation, and transcription sequencing (scNMT-seq)^85^ and single-cell nucleosome occupancy, DNA methylation, and RNA expression sequencing (scNOMeRe-seq)^86^ allow simultaneous methylation, chromatin accessibility, and transcriptome analysis. These techniques employ GpC methylase to detect the methylation of GpC (GCH) and CpG (WCG) sites. Although these methods involve physical separation of DNA and RNA, they provide valuable insights despite potential workflow complexities and costs^85,86^. A novel method, single-nucleus methylcytosine, chromatin accessibility, and transcriptome sequencing (snmCAT-seq), addresses these challenges by synthesizing cDNA using RT and methylated cytosine (mC)^87^ (Fig. 3c). This approach offers advantages for analyzing frozen samples and resolves mRNA poly(A) tail limitations in the nucleus^87^. These techniques were utilized to study developmental processes and the brain (Table 3).

Recently, Hi-C and RNA-seq were combined in HiRES, a technology that enables the simultaneous analysis of the transcriptome and chromatin conformation without physically separating RNA and DNA^88^. This approach was used to create a 3D genome and transcriptome atlas of postimplantation mouse embryos, uncovering genome-wide correlations between chromatin conformation and gene expression^88^. This novel omics combination has the potential to unravel developmental processes and gene expression patterns. Integrating additional epigenomic analyses, such as transcriptome analysis, into sn-m3C-seq could reveal relationships within different omics datasets for both normal and diseased samples.

### Proteome-related single-cell multimodal omics methods

The transcriptome serves as a proxy for the ‘proteome’. Proteins play crucial roles in defining the appearance, behavior, and response of cells. Although transcriptomics provides valuable insights into gene expression, it may not necessarily reflect the actual abundance of proteins within cells. Simultaneous transcriptome and proteome profiling within single cells enables researchers to bridge the gap between gene expression and protein abundance.

Cellular indexing of transcriptomes and epitopes by sequencing (CITE-seq)^89^ and RNA expression and protein sequencing (REAP-seq)^90^ combine highly multiplexed protein marker detection with unbiased conjugated transcriptome profiling. CITE-seq uses noncovalent streptavidin-biotinylated DNA barcodes, whereas REAP-seq uses covalent bonds between aminated DNA barcodes and antibodies. However, these methods focus on cell surface epitopes because of intracellular detection challenges.

Methods such as single-cell RNA and immunodetection (single-cell RAID)^91^ and single-cell protein and RNA coprofiling (SPARC)^92^ enable intracellular protein detection along with transcriptomics. Single-cell RAID leverages reversible fixation for intracellular immunostaining by using RNA barcode conjugates (ARCs)^91^. SPARC combines RNA sequencing with proximity extension assays for mRNA and intracellular protein measurements to overcome these limitations and the need for fixation^92^. Intranuclear cellular indexing of transcriptomes and epitopes (inCITE-seq) quantifies intranuclear proteins using DNA-conjugated antibodies and RNA sequencing on a droplet-based platform^93^.

Recent advances have enabled the simultaneous analysis of proteomes and transcriptomes. However, a lack of epigenomic analysis using these omics methods has left a disconnection in the flow of gene regulation. To overcome this flow disconnection, several multimodal omics technologies have been developed to analyze epigenomes.

To simultaneously analyze proteome and chromatin accessibility, several methods have been developed, including integrated cellular indexing of the chromatin landscape and epitopes (ICICLE-seq), ATAC with select antigen profiling by sequencing (ASAP-seq), and assay for transposase-accessible chromatin (PHAGE-ATAC)^94–96^. ICICLE-seq modifies permeabilized cell scATAC-seq to incorporate measurements using barcoded antibody reagents to capture epitopes^94^. ICICLE-seq utilizes the Tn5 transposome complex with capture sequences compatible with 10x Genomics 3′ scRNA-seq gel beads for chromatin accessibility and polyadenylated antibody barcode sequences for proteomes that can be selectively amplified^94^. ICICLE-seq has been extended to develop transcripts, epitopes, and accessibility sequencing (TEA-seq), which can simultaneously analyze the proteome, chromatin accessibility, and transcriptome using a droplet-based multimodal omics platform and incorporating scRNA-seq. Chromatin accessibility and proteome data can also be detected by ASAP-seq^95^. In addition, this method can detect not only surface proteins but also cellular proteins and mtDNA by extending mtscATAC-seq to incorporate antibodies conjugated with a poly(A) tail^95^. Similar to TEA-seq, ASAP-seq was further improved to allow for simultaneous analysis of the transcriptome by extending CITE-seq to enable compatibility with the 10x Genomics Multiome product^95^. The resulting method, DOGMA-seq, enabled trimodal analysis with the optional detection of mtDNA^95^. Another method, PHAGE-ATAC, utilizes phages for protein detection and can also analyze dual omics in a single cell by utilizing nanobody-displaying phages^96^. Phages are conjugated with a PAC tag, which is amplified using droplet linear PCR for analysis^96^. This method has been previously used to detect SARS-CoV-2 in human cell populations^96^.

Similarly, sequencing of nuclear protein epitope abundance, chromatin accessibility, and the transcriptome in single cells (NEAT-seq)^97^ has been developed to enable simultaneous quantification of nuclear protein epitope abundance, chromatin accessibility, and the transcriptome in single cells. This technique combines the principles of ATAC-Seq and RNA-Seq with nuclear protein quantification.

## Discussion

The development of single-cell-based methods is driven by the limitations of bulk cell-based approaches. By analyzing individual cells, researchers can reveal cellular heterogeneity and gain insights into various biological processes. However, these methods may not be sufficient to capture the complete picture of cellular reactions to diverse stimuli or conditions.

To address this limitation, single-cell multimodal omics techniques, which combine different single omics methods, have been developed and continue to improve. These approaches, such as sn-m3C-seq^64^, enable the examination of the relationships between different molecular features, such as DNA methylation, chromatin conformation, and gene expression. By integrating multiple omics datasets, researchers can obtain a deeper understanding of the complex mechanisms involved in individual cells.

However, most of these methods rely on next-generation sequencing (NGS), which typically produces short reads (up to 600 bp in length). This limitation poses challenges when analyzing long genomic regions or resolving complex genomic structures. Fortunately, long-read sequencing technologies have emerged as promising solutions.

Long-read sequencing technologies such as Oxford Nanopore Technologies (ONT) and PacBio single-molecule real-time (SMRT) sequencing can generate markedly longer reads than traditional NGS. These longer reads allow for improved characterization of genomic regions, including long-range interactions, structural variations, and multiple repeat regions. The integration of this technology into multiomics approaches holds great promise for overcoming the limitations associated with short-read sequencing.

By combining the strengths of single-cell-based methods, multiomics approaches, and long-read sequencing technologies, researchers can make new discoveries and gain a more comprehensive understanding of cellular processes and mechanisms. In the coming years, significant advancements in the field of multiomics toward the concept of ‘omniomics’, which aims to capture and characterize all molecules within a cell, are expected. The cellular phenome, which encompasses the full range of phenotypes expressed by a cell, serves as the goal of multiomics across various layers of biological information.
